# Identification of Loci Governing Agronomic Traits and Mutation Hotspots via a GBS-Based Genome-Wide Association Study in a Soybean Mutant Diversity Pool

**DOI:** 10.3390/ijms231810441

**Published:** 2022-09-09

**Authors:** Dong-Gun Kim, Jae Il Lyu, Jung Min Kim, Ji Su Seo, Hong-Il Choi, Yeong Deuk Jo, Sang Hoon Kim, Seok Hyun Eom, Joon-Woo Ahn, Chang-Hyu Bae, Soon-Jae Kwon

**Affiliations:** 1 Advanced Radiation Technology Institute, Korea Atomic Energy Research Institute, Jeongup 56212, Korea; 2 Research Center of Crop Breeding for Omics and Artificial Intelligence, Kongju National University, Yesan 32439, Korea; 3 Department of Horticultural Science, Chungnam National University, Daejeon 34134, Korea; 4 Department of Horticultural Biotechnology, Institute of Life Sciences & Resources, Kyung Hee University, Yongin 17104, Korea; 5 Department of Life Resources, Graduate School, Sunchon National University, Suncheon 57922, Korea

**Keywords:** soybean, genotyping-by-sequencing, genome-wide association study

## Abstract

In this study, we performed a genotyping-by-sequencing analysis and a genome-wide association study of a soybean mutant diversity pool previously constructed by gamma irradiation. A GWAS was conducted to detect significant associations between 37,249 SNPs, 11 agronomic traits, and 6 phytochemical traits. In the merged data set, 66 SNPs on 13 chromosomes were highly associated (FDR *p* < 0.05) with the following 4 agronomic traits: days of flowering (33 SNPs), flower color (16 SNPs), node number (6 SNPs), and seed coat color (11 SNPs). These results are consistent with the findings of earlier studies on other genetic features (e.g., natural accessions and recombinant inbred lines). Therefore, our observations suggest that the genomic changes in the mutants generated by gamma irradiation occurred at the same loci as the mutations in the natural soybean population. These findings are indicative of the existence of mutation hotspots, or the acceleration of genome evolution in response to high doses of radiation. Moreover, this study demonstrated that the integration of GBS and GWAS to investigate a mutant population derived from gamma irradiation is suitable for dissecting the molecular basis of complex traits in soybeans.

## 1. Introduction

Soybean (*Glycine max* L.), which is one of the most important agricultural crops worldwide, is a source of proteins, oils, carbohydrates, lipids, and essential minerals [1]. Soybeans are consumed directly or processed to produce edible oils and other food products for humans or feed products for animals [2]. In Korea, traditional food products derived from soybeans include tofu, soy flour, soymilk, soy sauce, and (fermented) soybean/red pepper paste [3]. Soybean is a rich source of phytochemicals and many types of functional components, including isoflavones, saponins, and fatty acids. These components are considered to have beneficial effects on human health (e.g., antioxidative effects) and may be useful for treating cancer [4].

Mutations are sudden genetic changes in the DNA of living cells that are not caused by genetic segregation or genetic recombination. Mutations induced by ionizing radiation range from simple base substitutions to single- and double-strand DNA breaks [5]. However, the use of crop varieties resulting from spontaneous mutations remains impractical because of the extensive selection required and the low mutation rates of only 10^−5^–10^−8^ per generation [6]. Mutation breeding refers to the purposeful application of mutations in plant breeding. Unlike hybridization and selection, mutation breeding can improve defects in elite cultivars without adversely affecting agronomic or quality-related characteristics [7]. Specifically, gamma irradiation can effectively induce genetic variations that alter many plant characteristics in a dose-dependent manner. Additionally, gamma rays produce mutant varieties directly, which is in contrast to the lengthy and laborious process required for conventional breeding [8]. There are currently 3365 mutant varieties of more than 210 plant species that have been registered for commercial use, including approximately 180 mutant soybean lines, which are in the FAO/IAEA mutant variety database (http://mvd.iaea.org accessed on 20 July 2022).

Mutation is a common phenomenon in organisms, and all gene sites are not equally mutable. The points that mutate at a higher frequency are often called “mutation hotspots” [9] and are valuable resources for exploring the mechanisms underlying mutation. Tan et al. [10] reported that the candidate gene *TMS5* of the T98S rice mutant irradiated with gamma radiation can convert cytosine (C) to adenine (A) in cds.71 to identify the mutation and act as a mutation hotspot. In addition, Xiong et al. [11] reported that wheat mutants identified mutant hotspot regions of chromosomes induced by gamma-rays and EMS at the transcriptome level. 

A single nucleotide polymorphism (SNP), which is a genetic variation at a specific nucleotide position of a genomic sequence among individuals, is usually caused by natural mutations and stresses (e.g., exposure to mutagens and tissue culturing) [12]. Additionally, SNPs can be categorized as either transitions (C/T or G/A) or transversions (C/G, A/T, C/A, or T/G), according to the nucleotide substitution. Moreover, SNP markers have been identified in numerous crop species, such as rice [13], maize [14], wheat [15], and soybean [16]. A few thousand markers are sufficient for quantitative trait locus mapping. Furthermore, genomic selection involves detecting and utilizing chromosomal intervals. Arrays with several thousand SNPs [17] and genotyping-by-sequencing (GBS) [18] are useful for these approaches.

Advances in next-generation sequencing technologies have aided the development of SNP detection methods. For example, GBS has recently emerged as a promising technique for simultaneously identifying SNPs and genotyping highly diverse species with large genomes [18]. This method relies on the digestion of genomic DNA with restriction enzymes and uses a pool of relatively large DNA fragments that are typically sequenced at low coverage. As a quick, extremely specific, and easily reproducible technique, GBS is a highly informative, high-throughput, and cost-effective tool for exploring plant genetic diversity on a genome-wide scale, and it requires no prior knowledge of the genome of a species of interest [18,19]. Accordingly, it has been successfully applied for the genotyping of diverse plant species, including soybean [20], barley [21], wheat [22], and maize [23]. In soybean, GBS, which enables the detection of many SNPs within a given population, has been used for genotyping [24]. Furthermore, it was recently used to detect SNPs, as well as small indels, in mutant soybean populations generated following proton beam [25] and fast neutron [26] treatments.

A genome-wide association study (GWAS), which is a powerful tool for identifying significant marker–trait associations, involves the detection of causative allelic variations at individual SNP markers related to a natural phenotypic variation. To conduct a GWAS, a large number of markers is required to provide adequate coverage of the entire genome. According to one estimate, tens of thousands of markers are necessary for the soybean genome [27]. Recent developments in high-throughput genotyping techniques, namely SNP genotyping arrays and GBS [20], have finally enabled researchers to obtain the required marker coverage for several hundred soybean lines. For soybean, genotyping involving either the Illumina BeadChip, or specific locus amplified fragment sequencing, has been combined with the GWAS approach to evaluate specific agronomic traits, including seed protein and oil concentrations [28], flowering time [29,30,31,32], flower color [33,34], node number, and seed coat color [35]. The GWAS method for dissecting complex traits has been successfully applied in studies of many plant species [36], including Arabidopsis [37], maize [38], and rice [39].

In a previous study, we constructed a mutant diversity pool (MDP) from 7000 gamma-irradiated soybean seeds and evaluated the genetic similarity among 208 soybean mutant lines using target region amplification polymorphism (TRAP) markers [40]. In the present study, we validated SNPs by GBS to identify mutations in 192 soybean MDP lines. We then conducted a GWAS to evaluate the association between SNPs and various agronomic and phytochemical traits.

## 2. Results

### 2.1. Characterization and Distribution of SNPs in the 192-MDP Soybean Genome

The GBS library constructed from 192 soybean MDP lines was sequenced using the Illumina HiSeq 2500 platform, which yielded approximately 940 million reads, with a mean quality score of 34.65 (Table S1). The demultiplexing of raw data and the removal of low-quality and adapter sequences were performed using GBSX. The number of raw reads varied from 13,098 (HK-32) to 11,360,994 (HK-15). The average Q30 was approximately 90.6%. The Q30 value for each line ranged from 88.2% (HK-mutant population) to approximately 92% (DB-mutant population) (Table S2). About 90% of the reads were successfully mapped to the soybean reference genome (Gmax_275_Wm82.a2.v1). The remaining unmapped reads, which originated from either the chloroplast or mitochondrial genomes, were mapped to more than one locus, or were removed on the basis of low map-quality scores (Table S3). Of the 978 million mapped reads, the number of reads distributed on each soybean chromosome varied from 37 million (Gm16) to 58 million (Gm18). The number of SNPs ranged from 1252 (Gm12) to 2877 (Gm18), whereas the number of Kb per SNP varied between 20.2 (Gm18) and 32.7 (Gm01). The number of SNPs per Mb of the entire genome ranged from 30.6 (Gm01) to 49.6 (Gm13 and 18), with an average of 38.5 (Table 1).

Finally, 37,673 SNPs were selected after applying the following filtering parameters: minimum depth = 5, minimum genotype quality = 20, and max-missing = 0.6 (40% missing data allowed). The average depth and average genome-wide transition/transversion ratio after filtering were approximately 22X and 1.6, respectively. The number of SNP transitions ranged from 16 (HK-32) to 12,456 (BS-25), whereas the number of SNP transversions varied from 6 (HK-32) to 7979 (BS-74) (Table S4). The SNPs in the MDP lines were functionally annotated using the reference genome sequence. Most of the SNPs (19,145; 50.86%) were located in genic regions, but a few were located in intergenic regions (7843; 20.80%). In terms of the genic region, the distribution of SNPs in the introns (5048; 13.40%), untranslated regions (UTRs) (1935; 5.15%), and coding sequences (CDSs) (6963 non-synonymous SNPs; 18.50%, and 5199 synonymous SNPs; 13.81%) was determined (Table S5).

### 2.2. Genetic Relationships and Population Structure

**Figure 1:**
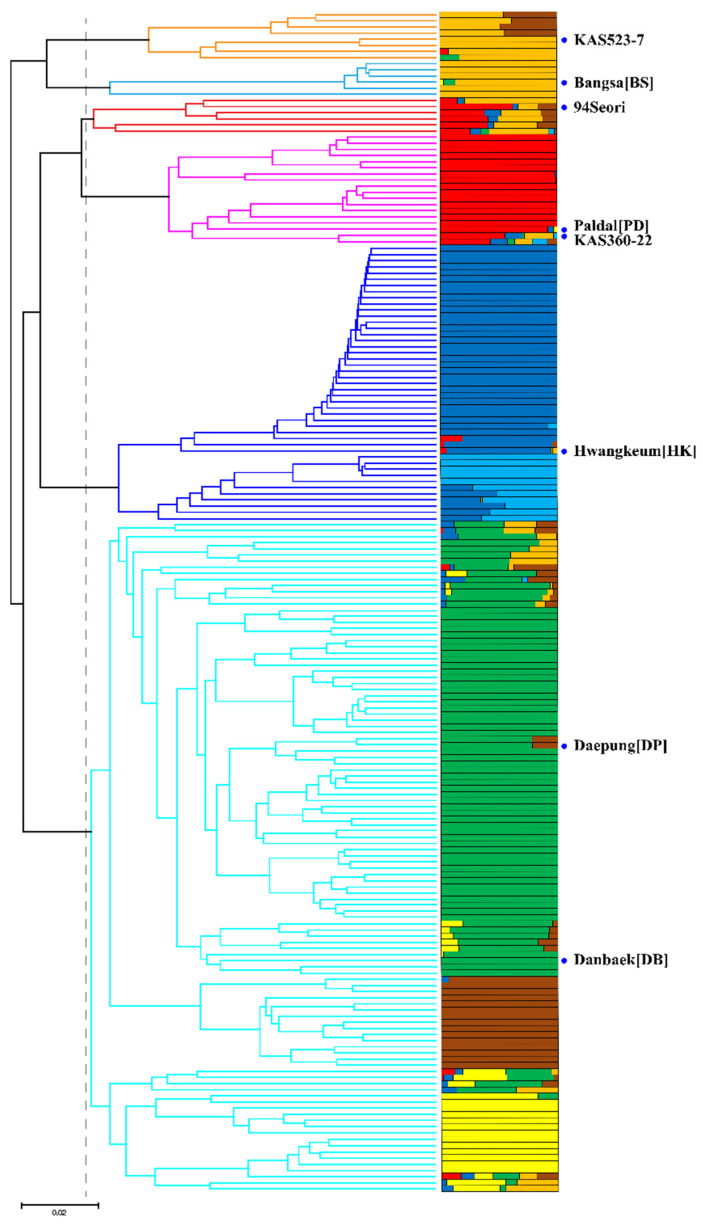
Genetic diversity and population structure of 192 soybean MDP lines. In the bar plot illustrating the population structure, each individual is represented by a single horizontal bar, with different colors used to represent different subpopulations.

A UPGMA-based dendrogram was constructed to clarify the genetic relationships among the 192 soybean MDP lines. At a genetic distance of 0.092, the 8 wild-type cultivars and their mutants were divided into approximately 6 major groups (Figure 1). Group 1 included 4 DB mutants, 2 DP mutants, 1 KAS523-7 mutant, and the wild-type KAS523-7. Group 2 comprised 5 BS mutants and the wild-type BS. Group 3 included 94Seori mutants and the wild-type 94Seori, along with 1 HK mutant. Group 4 comprised 15 PD mutants and the wild-type PD, as well as 1 KAS360-22 mutant and the wild-type KAS360-22. Group 5 included 44 HK mutants and the wild-type HK. Group 6, which was distinct from the other groups, consisted of 51 DP mutants, 56 DB mutants, and the wild-type DB and DP.

The population structure of the 192 soybean MDP lines, based on the genotypes acquired in this study, was analyzed using fastSTRUCTURE. Specifically, the population structure was assessed using *K* values ranging from 2 to 15 and the entire panel of high-quality SNPs. The estimated marginal likelihood was highest for *K* = 8. Each accession was assigned to one or more groups, depending on whether or not its genotype indicated it was admixed. The results of this analysis were consistent with the dendrogram topology. As denoted by different colors, the main membership composition of the eight wild-type lines in the corresponding groups was as follows: wild-type KAS523-7 in Group 1 was 100% orange, BS in Group 2 was 87% orange, 94Seori in Group 3 was 62% red, PD and KAS360-22 in Group 4 were 92% and 43% red, respectively, HK in Group 5 was 89% blue, and DB and DP in Group 6 were 78% and 100% green, respectively. When the dendrogram and the results of the population structure analysis were considered together, 96% of the 192 mutant lines (i.e., all except for four DB mutants, two DP mutants, and one HK mutant) were grouped with the corresponding wild-type line.

### 2.3. GWAS for Agronomic and Phytochemical Traits

The data for five qualitative (GT, FC, SCC, SHC, and SA) and six quantitative (DF, MD, SI, PH, NN, and RN) agronomic traits, as well as six phytochemical traits (TIC, PA, SAF, OA, LA, and ALA) for the 192 soybean MDP lines were obtained from earlier studies [40,41].

**Figure 2:**
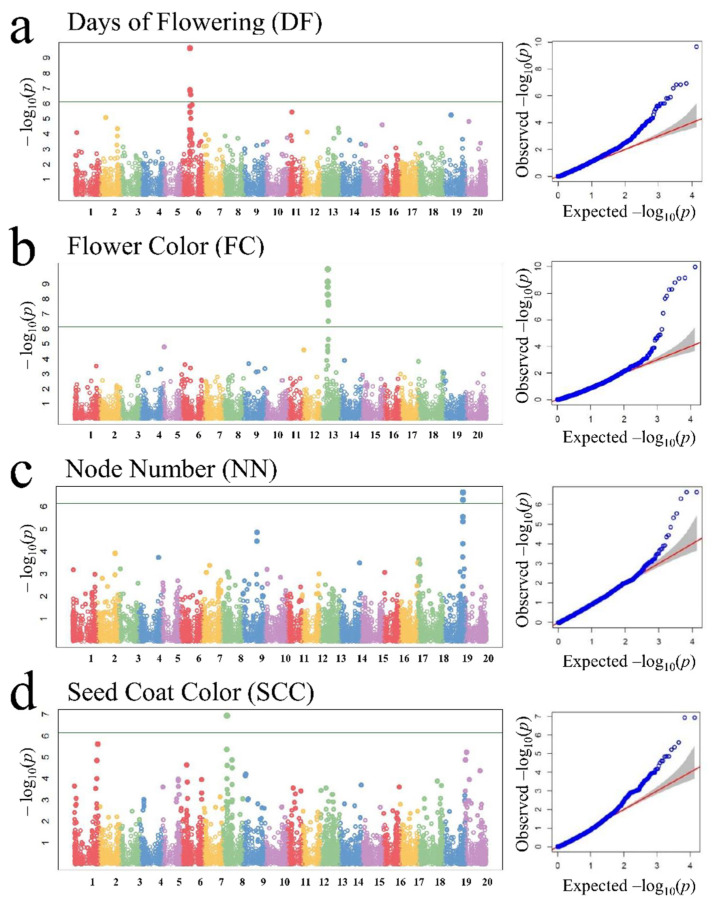
Manhattan and quantile–quantile plots generated by GAPIT to identify significant associations between SNP markers and phenotypic traits; (**a**): days of flowering (DF); (**b**): flower color (FC); (**c**): node number (NN); (**d**): seed coat color (SCC).

After removing the SNPs with a missing rate > 0.1, 37,249 of the 37,673 SNPs were selected for the GWAS analysis of the GBS merged dataset. According to the analysis, 66 SNPs on 13 chromosomes were highly associated (FDR *p* < 0.05) with the following four agronomic traits: DF (33 SNPs), FC (16 SNPs), NN (6 SNPs), and SCC (11 SNPs) (Table 2). The association analysis for DF revealed 33 significant marker–trait associations (SMTAs) on 10 chromosomes, including major associations on chromosome 6 (*p* = 2.20 × 10^−10^). One SMTA was detected on chromosomes 1 (*p* = 8.22 × 10^−^^5^), 7 (*p* = 0.00011), 11 (*p* = 3.58 × 10^−6^), 12 (*p* = 7.24 × 10^−5^), 15 (*p* = 2.50 × 10^−5^), and 20 (*p* = 1.52 × 10^−5^). Two SMTAs were detected on chromosomes 2 (*p* = 8.55 × 10^−6^) and 13 (*p* = 4.30 × 10^−5^), whereas three were detected on chromosome 19 (*p* = 5.62 × 10^−6^) (Table 2, Figure 2a). A total of 16 SNP markers on three chromosomes (5, 12, and 13) were highly associated (FDR *p* < 0.05) with FC. Fourteen major associations were detected on chromosome 13 (*p* = 1.02 × 10^−10^). One SMTA was detected on chromosomes 5 (*p* = 1.64 × 10^−5^) and 12 (*p* = 2.51 × 10^−5^) (Table 2, Figure 2b). The association analysis for NN detected six SMTAs on two chromosomes, including major associations on chromosome 5 (*p* = 2.37 × 10^−7^). One SMTA was detected on chromosome 9 (*p* = 1.40 × 10^−5^). The analysis also confirmed the presence of major SMTAs on chromosome 19 (45,317,378–45,367,407; *p* = 2.37 × 10^−7^) (Table 2, Figure 2c). Our analysis of markers associated with SCC revealed 11 SMTAs on three chromosomes, including major associations on chromosome 8. Two SMTAs were detected on chromosome 1 (*p* = 2.43 × 10^−6^), whereas three were detected on chromosome 20 (*p* = 5.92 × 10^−6^). We confirmed the presence of major SMTAs on chromosome 8 (9,589,829–21,840,533; *p* = 1.16 × 10^−7^) (Table 2, Figure 2d). In contrast, there were no markers highly associated with GT, MD, SHC, SI, SA, PH, RN, and TIC (i.e., FDR *p* ≥ 0.05) (Figure S1). Although there were no significant associations between SNPs and the four traits related to fatty acids (PA, OA, LA, and ALA), some SNPs were weakly associated with PA (chromosome 17) and OA (chromosome 18). The observed strong association between one SNP and SAF may have been a false positive (Figure S2).

### 2.4. Candidate Genes for Four Traits (DF, FC, NN, and SCC)

To identify candidate genes for DF, FC, NN, and SCC, the genes that were highly associated with SNPs (Table 2) were analyzed using the GBS data for the 192 soybean MDP lines (Table 3). A total of 39 genes revealed by GWAS were identified on chromosomes 1, 2, 6, 7, 8, 9, 11, 12, 13, 15, 19, and 20. For DF, 17 SNPs were located in 14 genes, of which 6 genes comprising 7 SNPs were on chromosome 6. The remaining 8 genes with 2, 1, 1, 1, 1, 3, and 1 SNPs were on chromosomes 2, 7, 11, 13, 15, 19, and 20, respectively. The 6 DF-related genes with 7 SNPs on chromosome 6 were identified as *Glyma.06g198100*, *Glyma.06g204600*, *Glyma.06g205600* (*RGP3* and *RGP*), *Glyma.06g205900* (*GAUT11*), *Glyma.06g208300* (*TET11*), and *Glyma.06g211600*. All of the SNPs were located in genic regions, including four and three nonsynonymous and synonymous SNPs, respectively. The locus most highly associated with DF, Chr06_19,461,588 (*p* = 2.20 × 10^−10^, R^2^ = 0.531) in *Glyma.06g205600*, was revealed to contain a nonsynonymous SNP. Specifically, compared with the wild-type sequence, T was replaced by C, which resulted in an amino acid change from Val (GUA) to Ala (GCA). For FC, the 13 detected SNPs were located in 13 different genes, of which the following 12 were on chromosome 13: *Glyma.13g070400*, *Glyma.13g070800*, *Glyma.13g070900* (*ALPHA-DOX1*, *DOX1*, *DIOX1*, and *PADOX-1*), *Glyma.13g071400* (*ATHSP22.0*), *Glyma.13g072000* (*SHT*), *Glyma.13g072600*, *Glyma.13g073400* (*MYB33* and *ATMYB33*), *Glyma.13g073500*, *Glyma.13g076300*, *Glyma.13g076800* (*EIN3* and *AtEIN3*), *Glyma.13g078500*, and *Glyma.13g078800* (Table 3). The locus most closely associated with FC, Chr13_17,554,641 (*p* = 1.02 × 10^−10^, R^2^ = 0.776) in *Glyma.13g073400*, contained a nonsynonymous SNP; the C-to-T change caused the amino acid to change from Ser (UCG) to Leu (UUG). The analysis of the SNPs in the candidate genes for NN indicated that all six SNPs were in genic regions (intron, two SNPs; 3′ UTR, two SNPs; and CDS, two nonsynonymous SNPs). For SCC, nine candidate genes were identified, among which the following five included six major SNPs and were detected on chromosome 8: *Glyma.08g124900* (*ZKT*), *Glyma.08g126500*, *Glyma.08g136600*, *Glyma.08g247500*, and *Glyma.08g249910* (*RGP2* and *ATRGP2*). These SNPs were mainly located in genic regions (CDS, two synonymous SNPs and two nonsynonymous SNPs; 3′ UTR and 5′ UTR, two SNPs).

## 3. Discussion

In this study, we identified SNPs in 192 soybean MDP lines using GBS data. Approximately 980 million reads were obtained, with 30.6 (Gm01) to 49.6 (Gm13 and 18) SNPs per Mb (average of 38.5) (Table 1). In an earlier study by Sonah et al. [33], in which SNPs were identified in 304 natural soybean accessions using a GBS pipeline, approximately 450 million reads were obtained, which is roughly half the number of reads generated in our study. Moreover, the SNP density for the population examined by Sonah et al. [30] ranged from 37 (Gm 01) to 59 (Gm 16) SNPs per Mb (average of 50). Although our average SNP density was lower, our minimum and maximum values for different chromosomes were similar to those of Sonah et al. [33]. One explanation for the lower SNP density in our study is that we used soybean mutant lines produced by the irradiation of cultivars. Accordingly, we may have assessed fewer subpopulations. Because GBS integrates the identification of molecular markers with the genotyping of large populations, it is ideal for plant breeding applications, even for species that lack a reference genome sequence or available polymorphism data. Earlier research confirmed that the GBS approach is suitable for soybean genetic analyses and marker development [20].

A previous examination of genetic diversity on the basis of 16 TRAP markers divided 208 soybean mutants into four groups [40]. Additionally, DB and DP mutants (along with the corresponding wild-type cultivars) were not clustered together in the phylogenetic tree. In contrast, both of these populations were included in Group 6 in the current study involving SNPs generated by GBS (Figure 1). This inconsistency between studies is probably because different genetic approaches were used in the two studies (i.e., GBS-generated markers developed on the basis of the soybean genome vs. TRAP markers developed from Arabidopsis and monoploid ESTs). Indeed, an analysis by Kim et al. [42] using 20 SSR markers developed from the soybean genome placed DB and DP lines in the same group as our study. Therefore, the wild-type DB and DP in Group 6 are closely related, with the common ancestor possibly being the material from which the cultivars were bred. Both DB [43] and DP [44] are popular soybean cultivars in Korea; however, we were unable to identify their common ancestor because of the limited availability of pedigree information for both cultivars, which were developed separately (DB in 1993 and DP in 2002).

In this study, we identified candidate genes and validated our GBS approach. A genome-wide association analysis was performed for 11 agronomic traits (GT, FC, SCC, SHC, SA, DF, MD, SI, PH, NN, and RN) and 6 phytochemical traits (TIC, PA, SAF, OA, LA, and ALA). A total of 66 SNPs on 13 chromosomes were highly associated (FDR *p* < 0.05) with DF (33 SNPs), FC (16 SNPs), NN (6 SNPs), and SCC (11 SNPs). We confirmed the existence of a major SMTA for DF on chromosome 6 (18,004,005–24,274,106; *p* = 2.20 × 10^−10^) (Figure 2a). In a GWAS of a natural population, Fang et al. [35] identified a similar SNP region (19,178,035–20,299,454; *p* = 7.08 × 10^−8^) on chromosome 6. Several studies of natural soybean populations [29,30,31,32,45] revealed an association between DF and chromosome 6 (Table 2). We also detected a major SMTA for FC on chromosome 13 (17,064,149–18,508,058; *p* = 1.02 × 10^−10^) (Figure 2b). This result is in accordance with the findings of earlier GWAS analyses of natural soybean accessions which indicated that FC is associated with the following regions on chromosome 13: 16,609,051–19,868,544 [35], 18,224,539 [34], and 2,514,518–4,818,964 [33] (Table 2). We also confirmed the existence of a major SMTA related to NN on chromosome 19 (45,317,378–45,367,407; *p* = 2.37 × 10^−7^) (Figure 2c). This SNP range corresponds to the SNP range (43,990,450–47,335,622; *p* = 5.89 × 10^−36^) on the same chromosome determined by Fang et al. [35] in a previous GWAS analysis of a natural population (Table 2). Finally, we confirmed that a major SMTA for SCC is present on chromosome 8 (9,589,829–21,840,533; *p* = 1.16 × 10^−7^) (Figure 2d), which is consistent with the SNP range (8,241,052–20,702,756; *p* = 1.20 × 10^−17^) on this chromosome identified during an earlier GWAS analysis of a natural population [34]. Another study involving natural populations [35] also detected the same SMTA on chromosome 8 (Table 2). However, we could not find FDR *p* < 0.05 in seven agronomic and six phytochemical traits (Figures S1 and S2). There are various reports of association results between traits and markers not identified in our study. Most of the markers regarding maturity days were reported on chromosome 16 [29,35], and those regarding plant height were reported on chromosome 19 [33,46]. Meng et al. [47] performed GWAS analysis on a Chinese soybean landrace population of 366 using RAD-seq and confirmed the association with isoflavones on chromosome 16. There are also many reports of the association of fatty acid contents. These reports confirmed that palmitic, stearic, oleic, linoleic, and linolenic acids were identified on chromosomes 5, 14, 20, 17, and 15, respectively [35,48,49,50]. In case of SMTAs, our results are consistent with those of earlier investigations that examined other genetic resources, including natural accessions and RIL populations. These observations suggest that genomic alterations occurred at the same loci in the gamma-irradiated mutants and in natural soybean populations. Generally, natural (spontaneous) mutations occur infrequently in higher plants, with only 10^−5^–10^−8^ per generation. There are a variety of reasons for this, including the fact plants are typically exposed to radiation (e.g., UV, X-ray, and other environmental radiation) at extremely low doses under natural conditions [6]. The relative rarity of natural mutations is also related to the evolution of plant genomes [51,52]. Many studies that applied whole-genome sequencing techniques have been conducted since Coulondre et al. [53] suggested the possibility of mutation hotspots. Recently, Tan et al. [10] reported that cds.71 in *TMS5* may be a mutation hotspot in the thermosensitive genic male sterile rice line T98S (induced by 300 Gy gamma irradiation). On the basis of transcriptome sequencing data, Xiong et al. [11] identified mutation hotspots on chromosome 1A (around the 50, 360, and 400 Mb positions) and the telomere of chromosomes 2A and 2B in four elite dwarf wheat mutants (*dm1*–*dm4*). Although all four of these mutants were derived from the winter cultivar Jing411, two were the result of a 250 Gy gamma irradiation (*dm1* and *dm2*) and two were generated following a 1–1.5% EMS treatment (*dm3* and *dm4*). In the current study, we compared the wild-type and mutant lines to detect the presence of 66 SNPs highly associated with specific traits in the mutants. Of the 20 SNPs associated with DF (chromosome 6; Chr06_19310425–Chr06_24274106), 19 were detected in all 15 PD mutants and in 39–49 of the 53 DP mutants. Similar results were obtained for the SNPs associated with FC, NN, and SCC. More specifically, all 5 BS mutants had 12 SNPs associated with FC; 29–50 of 60 DB mutants had 14 SNPs associated with FC; 12–15 of 15 PD mutants had 7 SNPs associated with FC; 7–13 of 53 DP mutants had 14 SNPs associated with FC; 1–2 of 4 94Seori mutants had 6 SNPs associated with FC; 25–29 of the DB mutants had 5 SNPs associated with NN; 6–7 of the PD mutants had 5 SNPs associated with NN; 34–35 of the DP mutants had 2 SNPs associated with NN; all BS mutants had 1 SNP associated with SCC; 2–10 of the DB mutants had 6 SNPs associated with SCC; and 1–6 of the DP mutants had 3 SNPs associated with SCC. However, in the HK mutants, the genomic regions corresponding to the 66 SNPs were relatively unaffected by the gamma irradiation, with only 4–5 of 45 mutants with 3 SNPs associated with FC, and only 1 mutant with 1 SNP associated with SCC. Accordingly, our findings may reflect the existence of mutation hotspots. These mutated loci might exhibit accelerated genome evolution in response to high gamma-ray doses. Indeed, all of the M_1_ seeds of the MDP lines were irradiated with 250 Gy gamma rays using the ^60^Co gamma irradiator when the soybean MDP was constructed [40].

In the present study, *Glyma.06g205600* (*RGP3* and *RGP*; *p* = 2.20 × 10^−10^, R^2^ = 0.531) on chromosome 6 was identified as a candidate gene for DF and was predicted to be highly correlated with the flowering time (Table 3). The protein encoded by this gene (i.e., RGP3) functions as a UDP-arabinose mutase that catalyzes the interconversion between the pyranose and furanose forms of UDP-L-arabinose. It is a reversibly autoglycosylated protein. Drakakai et al. [54] reported that mutations to RGPs result in abnormally enlarged vacuoles and poorly defined inner cell wall layers, leading to the development of abnormal pollen structures during pollen mitosis I. Zavliev et al. [55] revealed defects in plant development using transgenic tobacco plants expressing the Arabidopsis gene encoding class 1 reversibly glycosylated polypeptide 2 (*AtRGP2*); the flowering time of the transgenic lines was 1.5- to 2-times longer than that of the wild-type control. Moreover, the transgenic plants were stunted, with a rosette-like growth pattern, and their source leaves exhibited severe chlorosis, increased photoassimilate retention, and starch accumulation, which resulted in increased fresh and dry leaf weights. Thus, we speculate that the protein encoded by the candidate gene *Glyma.06g205600* modulates the flowering time because it adversely affects the pollen structure. Ambawat et al. [56] reported that MYB transcription factors affect plant development, cell shape and petal morphogenesis, cellular proliferation and differentiation, trichome development, phenylpropanoid metabolism, primary and secondary metabolism, and responses to hormones, biotic stress, light, and nutrient deficiency. Furthermore, anthocyanin accumulation in plants is positively and negatively regulated by MYB transcription factors [57,58]. Anthocyanin synthesis co-regulated by positive and negative regulatory factors is critical for flower coloration. The candidate gene *Glyma.13g073400* (*p* = 1.02 × 10^−10^, R^2^ = 0.776) associated with FC encodes MYB33. In Arabidopsis, AtMYB33 influences responses to hormones [59], as well as anther development (tapetum) and filament length [60].

Genome-wide association studies are useful for identifying genetic loci related to traits of interest [61]. The detected loci may be exploited in breeding programs by using marker-assisted breeding strategies. Additionally, GBS is a commonly used reliable and efficient technique [20]. In this study, SNP markers were identified in a population of soybean mutants on the basis of GBS data. A marker–trait association analysis involving 17 soybean traits was performed using GAPIT. In soybean, the estimated number of markers required to identify loci significantly affecting traits is in the tens of thousands [27]. In the current study, we obtained more than 37,000 markers, which is sufficient for detecting loci affecting various agronomic traits that are commonly targeted by breeding programs. The loci revealed as having a significant effect on a trait in the GBS analyses were detected in the merged GWAS analysis. Recently, an association analysis using universal SNP chips was performed for soybeans to identify SNPs associated with the fatty acid contents in 421 diverse accessions [62]. Completing a chip assay is an easy and convenient way to detect polymorphic markers, especially SNPs. Although we identified four SMTAs for DF, FC, NN, and SCC at previously reported loci, whether these associations involve the same SNPs remains to be determined. Mutation breeding using radiation results in the formation of new alleles via splicing and insertions/deletions [63]. Because most soybean SNP chips constructed to date [31,64] were generated using natural populations, their utility for analyzing mutants is unclear. Consequently, the GBS–GWAS method is the most suitable approach for investigating the genetic relationships in mutant soybean populations.

## 4. Materials and Methods

### 4.1. Plant Materials and Phenotyping

The 192 soybean MDP lines (184 mutants and 8 wild-type lines) used in this study were derived from 2 soybean landraces (KAS523-7 and KAS360-22) and 6 representative Korean soybean cultivars (‘94Seori’, ‘Bangsa’ [BS], ‘Paldal’ [PD], ‘Danbaek’ [DB], ‘Daepung’ [DP], and ‘Hwangkeum’ [HK]) from an earlier study [40]. The 192 soybean MDP lines in the M_12_ generation were phenotypically assessed for the following 11 agronomic traits: growth type (GT), flower color (FC), seed coat color (SCC), seed hilum color (SHC), and stem anthocyanin (SA) (i.e., qualitative traits); and days of flowering (DF), maturity days (MD), seed index (SI), plant height (PH), node number (NN), and ramification number (RN) (i.e., quantitative traits) (additional details are provided in Kim et al. [40]). The following six phytochemical traits were also analyzed: total isoflavone content (TIC) and the contents of five fatty acids, namely palmitic acid (PA; C16:0), stearic acid (SAF; C18:0), oleic acid (OA; C18:1), linoleic acid (LA; C18:2), and α-linolenic acid (ALA; C18:3) (additional details are provided in Kim et al. [41]).

### 4.2. DNA Extraction and GBS Analysis

Genomic DNA was extracted from the leaves of each mutant line using the DNeasy 96 Plant kit (Qiagen, Leipzig, Germany). After quantifying the extracted DNA using the NanoDrop ND-1000 spectrophotometer (Thermo Fisher Scientific, Waltham, MA, USA), the DNA concentrations were adjusted to 50–100 ng/µL (total of 30–50 µL per sample) for the GBS analysis.

A GBS approach was used for SNP genotyping. Specifically, a GBS library was prepared by digesting the DNA of individual soybean plants with *Ape*KI (New England Biolabs, Ipswich, MA, USA). Bar-coded adapters were then ligated to the digested fragments. The 192 barcode sequences (4–8 nucleotides long) used for tagging the samples are listed in Supplementary Table S6. The appropriate adapter concentration was determined and used to construct the library according to the GBS protocol, with minor modifications, as described in Elshire et al. [18]. Finally, the GBS library was sequenced using the HiSeq 2500 high-throughput sequencing platform (Illumina, San Diego, CA, USA). The GBS raw read data are available in the NCBI Sequence Read Archive (accession PRJNA845013). Demultiplexing was performed using the barcode sequences. Additionally, adapter sequences were removed, and low-quality sequences were trimmed using GBSX software (v1.3, Leuven, Belgium) [65]. The retained clean reads for each sample were aligned to the reference genome (Gmax_275_Wm82.a2.v1; Schmutz et al. [66]) using the Burrows–Wheeler Aligner software (v0.7.17, Hinxton, UK, Li and Durbin [67]), and then the SAMtools software (v1.7.6, Hinxton, UK) [68] was used to convert the alignment files to BAM files. If multiple read pairs had identical external coordinates, then only the pair with the highest mapping quality was retained. Variant calling was performed for all samples using the Genome Analysis Toolkit software (v3.8.0, Cambridge, MA, USA, McKenna et al. [69]). The following parameters of Vcftools (v0.1.16, Hinxton, UK) [70] were used to filter SNPs: minimum depth = 5, minimum genotype quality = 20, and max-missing = 0.6 (40% missing data allowed). The SNPs were functionally and structurally annotated using the SnpEff tool (v4.3, Detroit, MI, USA) [71] and the annotated soybean genome available in the Phytozome database [72].

### 4.3. Genetic Diversity and Population Structure Analyses

Genetic relationships were investigated by constructing a UPGMA-based dendrogram using TASSEL software (v5.2.17, Ithaca, NY, USA) [73]. The population structure of the 192 soybean MDP lines was analyzed using fastSTRUCTURE [74]. The SNP genotype data for the 192 soybean MDP lines were converted to the variant call format, and the fastSTRUCTURE analysis was performed with *K* = 2… 15. This program has been widely used to calculate posterior inference on the basis of the Bayesian framework [75]. A phylogenetic analysis of the 192 soybean MDP lines was performed using the GBS data.

### 4.4. GWAS Analysis

To ensure sufficient evaluation of the genetic diversity of the 192 soybean MDP lines, 17 phenotypic traits, comprising 5 qualitative traits (GT, FC, SCC, SHC, and SA), 6 quantitative traits (DF, MD, SI, PH, NN, and RN), and 6 phytochemical traits (TIC, PA, SAF, OA, LA, and ALA) were included in the GWAS analysis. After filtering SNPs with <10% missing data, 37,249 SNPs were selected and used for the GWAS, which was performed using a compressed mixed linear model [76]. All analyses were conducted using the Genomic Association and Prediction Integrated Tool (GAPIT; Lipka et al. [77]) in the R program.

## 5. Conclusions

In summary, we previously constructed an MDP core collection that reflects the diversity among soybean lines in terms of agronomic traits. We focused on identifying loci that were identical to the genetic loci in existing natural populations. We revealed substantial variations in 17 soybean agronomic traits, including 6 phytochemical traits, and demonstrated the utility of GWAS for detecting genetic factors underlying important agronomic traits. The genetic basis of these traits was then dissected in an association analysis using 37,249 SNPs obtained by GBS. A total of 66 SNPs on 13 chromosomes were determined to be highly associated with the examined traits. All of the major SNP markers for four agronomic traits (DF, FC, NN, and SCC) were detected at the same genomic loci in the mutants and the natural populations. The soybean MDP core collection is a useful resource for future research on soybean genetic diversity, as well as soybean mutation breeding. Furthermore, the SNPs described herein may be applicable as new markers in future soybean studies.

## Figures and Tables

**Table 1:** Chromosomal distribution and frequency of SNPs identified using the GBS approach in 192 soybean MDP lines.

Chromosome	Length (bp)	No. of SNPs	Kbs/SNP	SNPs/Mb
Gm01	56,831,624	1738	32.7	30.6
Gm02	48,577,505	1719	28.3	35.4
Gm03	45,779,781	1619	28.3	35.4
Gm04	52,389,146	1884	27.8	36.0
Gm05	42,234,498	1532	27.6	36.3
Gm06	51,416,486	2155	23.9	41.9
Gm07	44,630,646	1627	27.4	36.5
Gm08	47,837,940	2092	22.9	43.7
Gm09	50,189,764	1852	27.1	36.9
Gm10	51,566,898	1935	26.6	37.5
Gm11	34,766,867	1323	26.3	38.1
Gm12	40,091,314	1252	32.0	31.2
Gm13	45,874,162	2277	20.1	49.6
Gm14	49,042,192	1949	25.2	39.7
Gm15	51,756,343	2025	25.6	39.1
Gm16	37,887,014	1801	21.0	47.5
Gm17	41,641,366	1915	21.7	46.0
Gm18	58,018,742	2877	20.2	49.6
Gm19	50,746,916	1799	28.2	35.5
Gm20	47,904,181	1878	25.5	39.2
Scaffolds	29,311,887	424	69.1	14.5
Total	978,495,272	37,673	26.0	38.5

**Table 2:** Details regarding the loci associated with different traits revealed by a GWAS of 192 soybean MDP lines.

Traits	Total SNPs	Chr. No.	Significant Region		*p*-Value	Chr. No.	Regions		*p*-Value	References
			Start	End			Start	End		
DF	20	6	18,004,005	24,274,106	2.20 × 10^−^^10^	6	6,077,874	16,773,415	1.50 × 10^−^^7^	[28]
	1	1		3,427,092	8.22 × 10^−5^	6	12,336,492	12,336,709	0.00589	[27]
	2	2	13,487,773	40,934,117	8.55 × 10^−6^	6	2,104,472	2,108,449		[42]
	1	7		4,104,188	0.00011	6	19,178,035	20,299,454	7.08 × 10^−8^	[32]
	1	11		9,299,855	3.58 × 10^−6^	6	23,848,501	46,820,673		[29]
	1	12		9,786,525	7.24 × 10^−5^	6	19,919,551	20,263,848		[26]
	2	13	41,504,580	42,826,870	4.30 × 10^−5^					
	1	15		48,448,735	2.50 × 10^−5^					
	3	19	18,391,540	18,391,588	5.62 × 10^−6^					
	1	20		8,663,045	1.52 × 10^−5^					
FC	14	13	17,064,149	18,508,058	1.02 × 10^−^^10^	13	16,609,051	19,868,544	6.76 × 10^−^^166^	[32]
	1	12		1,967,332	2.51 × 10^−5^	13		18,224,539	4.89 × 10^−^^29^	[31]
	1	5		2,807,049	1.64 × 10^−5^	13	2,514,518	4,818,964	3.39 × 10^−^^17^	[30]
NN	5	19	45,317,378	45,367,407	2.37 × 10^−7^	19	43,990,450	47,335,622	5.89 × 10^−^^36^	[32]
	1	9		33,307,361	1.40 × 10^−5^					
SCC	6	8	9,589,829	21,840,533	1.16 × 10^−7^	8	7,800,853	9,079,037	2.63 × 10^−^^35^	[32]
	3	20	444,347	33,593,731	5.92 × 10^−6^	8	8,241,052	20,702,756	1.20 × 10^−^^17^	[31]
	2	1	51,636,235	53,677,289	2.43 × 10^−6^					

DF: days of flowering; FC: flower color; NN: node number; SCC: seed coat color; Chr. No.: chromosome number.

**Table 3:** Candidate genes and SNPs significantly associated with four traits (DF, FC, NN, and SCC) on the basis of the GWAS results.

Traits	Candidate Gene	Lead SNP	Allele	Location Site	*p*-Value	R^2^	Symbols
DF	*Glyma.02g130700*	Chr02_13487773	A/C	Intron	8.55 × 10^−6^	0.439	ATPPC4, PPC4
	*Glyma.02g221900*	Chr02_40934117	A/G	Nonsynonymous	4.64 × 10^−5^	0.501	
	*Glyma.06g198100*	Chr06_18004005	C/T	Synonymous	5.24 × 10^−5^	0.512	
	*Glyma.06g204600*	Chr06_19310425	T/C	Nonsynonymous	0.0001	0.507	
		Chr06_19315351	A/T	Nonsynonymous	3.87 × 10^−6^	0.526	
	*Glyma.06g205600*	Chr06_19461588	T/C	Nonsynonymous	2.20 × 10^−10^	0.531	RGP3, RGP
	*Glyma.06g205900*	Chr06_19677827	T/A	Nonsynonymous	1.21 × 10^−7^	0.499	GAUT11
	*Glyma.06g208300*	Chr06_20321899	C/T	Synonymous	6.57 × 10^−5^	0.529	TET11
	*Glyma.06g211600*	Chr06_21142419	C/T	Synonymous	2.65 × 10^−7^	0.520	
	*Glyma.07g048500*	Chr07_4104188	C/T	Synonymous	0.0001	0.485	LHY, LHY1
	*Glyma.11g121700*	Chr11_9299855	C/T	Downstream	3.58 × 10^−6^	0.489	GLB1, AHB1, ARATH GLB1, NSHB1, ATGLB1, HB1
	*Glyma.13g320800*	Chr13_41504580	A/C	UTR5	4.30 × 10^−5^	0.423	ATMGT10, GMN10, MGT10, MRS2-11
	*Glyma.15g255200*	Chr15_48448735	G/A	Synonymous	2.50 × 10^−5^	0.465	
	*Glyma.19g066800*	Chr19_18391540	T/G	Intron	5.62 × 10^−6^	0.496	ATX4, SDG16
		Chr19_18391584	A/G	Intron	5.62 × 10^−6^	0.496	ATX4, SDG16
		Chr19_18391588	T/C	Intron	5.62 × 10^−6^	0.496	ATX4, SDG16
	*Glyma.20g046800*	Chr20_8663045	G/A	Downstream	1.52 × 10^−5^	0.441	ATCDPMEK, PDE277, ISPE, CDPMEK
FC	*Glyma.12g027300*	Chr12_1967332	C/A	Downstream	2.51 × 10^−5^	0.730	MOD1, ENR1
	*Glyma.13g070400*	Chr13_17064149	G/A	Intron	7.48 × 10^−10^	0.769	
	*Glyma.13g070800*	Chr13_17112561	C/T	Downstream	1.42 × 10^−5^	0.732	
	*Glyma.13g070900*	Chr13_17120843	C/T	Nonsynonymous	5.00 × 10^−9^	0.761	ALPHA-DOX1, DOX1, DIOX1, PADOX-1
	*Glyma.13g071400*	Chr13_17168452	A/C	Nonsynonymous	1.59 × 10^−9^	0.766	ATHSP22.0
	*Glyma.13g072000*	Chr13_17304314	T/C	Synonymous	6.75 × 10^−10^	0.769	SHT
	*Glyma.13g072600*	Chr13_17394477	G/C	Downstream	3.41 × 10^−5^	0.729	
	*Glyma.13g073400*	Chr13_17554641	C/T	Nonsynonymous	1.02 × 10^−10^	0.776	MYB33, ATMYB33
	*Glyma.13g073500*	Chr13_17622554	A/T	Nonsynonymous	5.22 × 10^−9^	0.761	
	*Glyma.13g076300*	Chr13_18038564	G/C	Synonymous	5.02 × 10^−6^	0.736	
	*Glyma.13g076800*	Chr13_18150461	A/T	Intron	1.57 × 10^−8^	0.757	EIN3, AtEIN3
	*Glyma.13g078500*	Chr13_18457847	T/C	Synonymous	2.54 × 10^−8^	0.755	
	*Glyma.13g078800*	Chr13_18508058	C/T	Intron	3.14 × 10^−7^	0.746	
NN	*Glyma.09g133900*	Chr09_33307361	G/A	Nonsynonymous	1.40 × 10^−5^	0.423	
	*Glyma.19g196000*	Chr19_45317378	G/C	Nonsynonymous	2.84 × 10^−6^	0.434	SPY
		Chr19_45322411	C/T	Intron	4.80 × 10^−6^	0.430	SPY
		Chr19_45326559	A/C	Intron	5.03 × 10^−7^	0.448	SPY
	*Glyma.19g196500*	Chr19_45367388	A/G	UTR3	2.37 × 10^−7^	0.453	emb2735
		Chr19_45367407	T/C	UTR3	2.37 × 10^−7^	0.453	emb2735
SCC	*Glyma.01g180100*	Chr01_51636235	G/A	Synonymous	1.44 × 10^−5^	0.506	
	*Glyma.01g203600*	Chr01_53677289	C/A	Downstream	2.43 × 10^−6^	0.517	
	*Glyma.08g124900*	Chr08_9589829	A/G	Nonsynonymous	4.37 × 10^−6^	0.513	ZKT
	*Glyma.08g126500*	Chr08_9744316	G/A	Synonymous	1.16 × 10^−7^	0.537	
		Chr08_9744418	T/A	Synonymous	1.16 × 10^−7^	0.537	
	*Glyma.08g136600*	Chr08_10455379	A/T	UTR5	2.38 × 10^−5^	0.503	
	*Glyma.08g247500*	Chr08_21445554	A/G	Nonsynonymous	1.39 × 10^−5^	0.506	
	*Glyma.08g249900*	Chr08_21840533	C/T	UTR3	3.23 × 10^−5^	0.501	RGP2, ATRGP2
	*Glyma.20g024800*	Chr20_2681018	A/T	Synonymous	5.92 × 10^−6^	0.511	
	*Glyma.20g092500*	Chr20_33593731	T/G	Nonsynonymous	4.33 × 10^−5^	0.499	

DF: days of flowering; FC: flower color; NN: node number; SCC: seed coat color; Candidate gene: a plausible biological candidate gene in the locus or the nearest annotated gene to the SNP; Chr.: the chromosome on which the corresponding locus is located; Allele: the information in corresponding columns are based on the SNP; R^2^ indicates the phenotypic variance explained by the SNP marker.

## Data Availability

The GBS raw reads can be accessed from the NCBI website (BioProject number PRJNA845013).

## Supplementary Materials

The supporting information can be downloaded at: https://www.mdpi.com/article/10.3390/ijms231810441/s1.
