# Supplementary material for: Identification of Loci Governing Agronomic Traits and Mutation Hotspots via a GBS-Based Genome-Wide Association Study in a Soybean Mutant Diversity Pool
Source: Int J Mol Sci. 2022 Sep 9;23(18):10441. doi: 10.3390/ijms231810441 (PMC9499481; doi:10.3390/ijms231810441)
Supplement: Supplementary file 1 [file ijms-23-10441-s001.zip › Table S1.pdf]

Table S1. Information regarding the nucleotide sequence reads in the GBS library.

	Yields (bp)	Reads	%, of >= Q30 Bases	Mean Quality Score
GBS_1_F	22,642,904,978	224,187,178	91.57	34.89
GBS_1_R	22,642,904,978	224,187,178	91.28	34.88
GBS_2_F	24,860,207,569	246,140,669	89.55	34.47
GBS_2_R	24,860,207,569	246,140,669	88.49	34.37
Total	95,006,225,094	940,655,694		
Mean			90.22	34.65
