# Supplementary material for: Identification of Loci Governing Agronomic Traits and Mutation Hotspots via a GBS-Based Genome-Wide Association Study in a Soybean Mutant Diversity Pool
Source: Int J Mol Sci. 2022 Sep 9;23(18):10441. doi: 10.3390/ijms231810441 (PMC9499481; doi:10.3390/ijms231810441)
Supplement: Supplementary file 1 [file ijms-23-10441-s001.zip › Table S2.pdf]

Table S2. Sequencing summary.

No.	Lines	Total read bases (bp)	Total reads	Q20 (%)	Q30 (%)
1	KAS523-7	726,119,557	7,649,550	95.86	91.69
2	KAS523-7-Ru	435,102,753	4,581,976	95.97	91.88
3	94Seori	370,232,500	3,903,690	95.99	91.93
4	JoseangSeori	176,410,736	1,857,636	96.18	92.27
5	J-S	701,348,083	7,391,774	96.16	92.22
6	J-D01	679,102,664	7,165,048	95.94	91.81
7	J-D02	819,499,520	8,628,962	96.11	92.15
8	KAS360-22	493,564,235	5,195,146	95.95	91.86
9	KAS360-22-W	684,556,560	7,201,464	96.23	92.31
10	BangSa	966,985,012	10,288,844	95.96	91.75
11	BS-25	715,907,775	7,547,426	96.00	91.95
12	BS-63	310,271,146	3,275,656	96.05	92.00
13	BS-73	646,501,528	6,821,852	95.81	91.64
14	BS-74	882,215,094	9,307,644	95.92	91.82
15	BS-84	843,593,595	8,899,140	95.95	91.86
16	PalDal	651,658,366	6,872,236	96.21	92.34
17	PD-D01	793,443,985	8,377,176	95.64	91.28
18	PD-D02	687,974,690	7,260,034	96.12	92.09
19	PD-D03	526,273,268	5,564,744	95.93	91.82
20	PD-D04	245,232,233	2,595,514	95.86	91.72
21	PD-D05	531,029,455	5,608,760	96.08	92.07
22	PD-D06	264,235,694	2,788,036	95.92	91.83
23	PD-D07	688,202,488	7,262,964	95.85	91.66
24	PD-I01	430,206,525	4,531,986	96.30	92.46
25	PD-I02	284,633,528	3,004,114	96.15	92.19
26	PD-I03	778,676,362	8,214,314	96.11	92.16
27	PD-I04	713,289,707	7,530,858	95.88	91.78
28	PD-I05	379,618,173	4,006,582	96.29	92.44
29	PD-I06	636,509,846	6,725,074	96.13	92.19
30	PD-I07	341,510,489	3,605,464	96.14	92.20
31	PD-I08-W	455,624,438	4,815,088	95.87	91.70
32	DanBaek	629,626,375	6,642,424	95.85	91.69
33	DB-003	849,483,148	8,963,300	95.78	91.58
34	DB-004	721,068,799	7,623,078	95.93	91.80
35	DB-005	654,696,959	6,924,202	96.14	92.23
36	DB-006	316,693,634	3,355,652	95.87	91.69
37	DB-007	295,156,939	3,122,512	96.13	92.16
38	DB-008	369,626,693	3,910,016	95.85	91.69

39	DB-009	473,347,593	5,010,838	96.01	92.00
40	DB-010	374,150,266	3,952,226	95.81	91.63
41	DB-016	263,022,010	2,779,016	96.10	92.12
42	DB-019	637,224,785	6,737,314	96.04	92.00
43	DB-024	206,209,693	2,181,262	96.01	91.93
44	DB-026	263,169,518	2,780,794	96.10	92.14
45	DB-027	492,582,468	5,208,832	96.06	92.02
46	DB-029	350,702,093	3,713,012	95.84	91.66
47	DB-030	540,479,813	5,710,576	96.03	92.03
48	DB-031	564,023,166	5,959,672	95.75	91.49
49	DB-033	243,305,777	2,570,600	95.77	91.56
50	DB-034	409,970,543	4,334,532	96.02	91.97
51	DB-035	130,236,862	1,377,770	96.19	92.28
52	DB-036	362,408,800	3,831,858	95.98	91.93
53	DB-037	194,697,139	2,058,634	96.04	92.01
54	DB-038	229,586,415	2,429,596	95.95	91.85
55	DB-039	249,012,384	2,631,398	95.90	91.77
56	DB-040	614,046,624	6,503,692	95.39	90.80
57	DB-041	435,634,186	4,609,410	95.75	91.52
58	DB-044	560,288,475	5,930,368	96.10	92.11
59	DB-045	629,527,106	6,666,282	96.25	92.40
60	DB-046	350,850,783	3,720,432	96.00	91.96
61	DB-049	645,308,789	6,849,680	96.05	92.02
62	DB-050	385,997,699	4,104,760	96.10	92.11
63	DB-051	592,175,833	6,283,082	96.03	91.98
64	DB-054	568,865,252	6,017,742	96.03	91.97
65	DB-056	510,357,858	5,394,972	95.97	91.87
66	DB-059	574,086,191	6,081,320	95.94	91.87
67	DB-060	390,990,164	4,142,840	96.00	91.91
68	DB-061	404,795,142	4,284,184	96.06	92.03
69	DB-062	311,737,629	3,300,622	96.08	92.11
70	DB-063	439,013,118	4,644,350	95.94	91.88
71	DB-064	256,351,843	2,715,172	95.81	91.62
72	DB-065	442,352,683	4,675,970	95.92	91.78
73	DB-067	473,018,538	5,012,164	95.92	91.81
74	DB-068	332,061,389	3,517,866	95.85	91.63
75	DB-072	204,015,302	2,159,018	96.09	92.12
76	DB-073	122,059,534	1,291,784	95.64	91.32
77	DB-074	130,817,825	1,390,340	95.73	91.42
78	DB-075	2,125,912	22,656	93.06	87.71
79	DB-076	555,443,279	5,893,780	95.88	91.71

80	DB-078	503,184,963	5,333,564	95.92	91.76
81	DB-079	156,227,906	1,657,188	95.89	91.75
82	DB-080	520,443,700	5,529,720	95.72	91.42
83	DB-083	2,565,657	28,150	96.01	91.83
84	DB-085	260,918,135	2,771,102	96.00	91.97
85	DB-086	400,739,718	4,248,948	95.71	91.44
86	DB-087	175,502,404	1,858,618	96.15	92.20
87	DB-088	145,810,809	1,550,036	95.93	91.80
88	DB-089	160,478,838	1,697,242	95.84	91.71
89	DB-090	387,343,216	4,099,704	95.94	91.84
90	DB-091	157,394,392	1,670,114	95.70	91.41
91	DB-092	334,422,275	3,542,512	95.60	91.25
92	DB-093	115,099,416	1,219,316	95.72	91.45
93	DaePung	245,020,667	2,596,786	96.01	91.96
94	DP-009	242,448,070	2,569,880	95.86	91.64
95	DP-012	209,435,188	2,223,770	95.74	91.46
96	DP-027	168,470,605	1,782,038	95.58	91.16
97	DP-028	992,894,560	10,441,632	94.76	89.44
98	DP-029	370,434,052	3,899,686	94.82	89.51
99	DP-046	390,270,386	4,114,452	94.88	89.64
100	DP-048	185,041,446	1,948,254	95.09	89.99
101	DP-051	707,207,926	7,443,956	95.13	90.08
102	DP-052	703,387,718	7,406,186	94.77	89.42
103	DP-053	685,711,200	7,231,542	94.96	89.76
104	DP-054	398,353,803	4,196,558	94.78	89.45
105	DP-055	949,574,657	9,988,236	95.13	90.01
106	DP-056	884,152,671	9,310,106	95.08	89.97
107	DP-057	910,485,807	9,590,708	94.97	89.83
108	DP-059	316,686,697	3,339,232	94.89	89.63
109	DP-060	762,602,942	8,054,884	94.56	89.12
110	DP-061	974,181,401	10,272,800	94.87	89.66
111	DP-062	659,134,911	6,963,660	94.93	89.74
112	DP-079	587,698,457	6,195,362	95.22	90.29
113	DP-080	819,196,246	8,634,364	94.53	89.03
114	DP-081	1,056,217,121	11,155,920	95.00	89.74
115	DP-083	861,282,059	9,085,478	94.72	89.35
116	DP-084	223,802,293	2,361,404	94.68	89.34
117	DP-085	731,461,863	7,718,404	94.89	89.65
118	DP-086	341,202,047	3,599,386	94.72	89.37
119	DP-087	536,502,924	5,658,186	94.71	89.34
120	DP-090	373,106,353	3,932,926	95.26	90.29

121	DP-091	277,529,291	2,923,242	95.03	89.89
122	DP-092	782,526,762	8,248,144	95.05	89.98
123	DP-093	855,822,774	9,040,980	94.73	89.40
124	DP-094	274,494,907	2,897,298	95.20	90.17
125	DP-095	779,778,877	8,236,086	94.97	89.78
126	DP-097	275,199,979	2,900,834	95.09	90.04
127	DP-106	448,354,394	4,731,178	94.73	89.38
128	DP-111	599,167,688	6,318,296	94.81	89.55
129	DP-114	811,160,285	8,550,804	94.57	89.18
130	DP-117	358,581,180	3,814,786	94.79	89.39
131	DP-120	615,826,172	6,507,376	95.03	89.95
132	DP-121	154,917,321	1,645,238	94.78	89.41
133	DP-127	193,808,425	2,063,314	95.08	89.90
134	DP-129	174,054,348	1,852,330	94.69	89.26
135	DP-131	259,533,335	2,762,026	95.04	89.91
136	DP-132	262,776,634	2,793,136	94.65	89.19
137	DP-140	333,635,289	3,519,294	95.01	89.89
138	DP-152	627,360,681	6,625,840	94.96	89.78
139	DP-172	203,915,901	2,153,298	94.88	89.62
140	DP-178	307,141,558	3,249,752	95.04	89.93
141	DP-179	499,605,950	5,295,250	94.98	89.76
142	DP-183	389,973,923	4,118,472	94.68	89.32
143	DP-184	759,031,850	8,012,342	94.84	89.61
144	DP-190	626,768,006	6,614,588	94.49	88.93
145	DP-192	396,026,387	4,175,284	94.57	89.14
146	DP-200	563,428,161	5,961,044	94.88	89.63
147	HwangKeum	149,675,280	1,589,458	95.10	90.00
148	HK-1	637,202,292	6,735,810	94.76	89.43
149	HK-2	338,472,419	3,576,926	94.83	89.52
150	HK-3	277,170,609	2,942,544	94.88	89.61
151	HK-4	348,311,966	3,678,100	94.58	89.09
152	HK-5	731,734,994	7,742,168	94.16	88.31
153	HK-6	573,733,843	6,115,882	94.59	89.10
154	HK-8	498,890,770	5,305,742	95.04	89.86
155	HK-9	750,140,677	7,941,772	95.20	90.20
156	HK-10	409,626,589	4,334,910	94.86	89.63
157	HK-11	693,108,784	7,343,738	95.01	89.88
158	HK-12	352,812,210	3,738,022	95.07	89.98
159	HK-13	604,358,812	6,396,266	95.03	89.92
160	HK-14	704,987,253	7,448,398	94.84	89.56
161	HK-15	1,071,310,458	11,360,994	94.80	89.42

162	HK-17	592,091,099	6,270,054	94.81	89.55
163	HK-18	379,454,541	4,039,860	95.04	89.86
164	HK-19	436,134,846	4,621,662	94.95	89.71
165	HK-20	517,027,332	5,461,650	94.95	89.79
166	HK-23	558,860,106	5,906,542	94.70	89.36
167	HK-24	198,827,537	2,109,692	94.69	89.30
168	HK-25	505,677,536	5,357,086	94.71	89.29
169	HK-27	454,286,427	4,808,426	94.76	89.43
170	HK-28	398,781,913	4,217,144	94.68	89.28
171	HK-29	220,104,536	2,323,074	94.93	89.75
172	HK-30	139,967,339	1,483,822	94.45	88.88
173	HK-31	196,051,864	2,073,644	94.47	88.90
174	HK-32	1,232,816	13,098	88.92	80.39
175	HK-33	683,866,305	7,246,082	94.65	89.20
176	HK-34	553,971,293	5,859,472	94.74	89.37
177	HK-36	225,902,308	2,396,952	94.79	89.48
178	HK-37	475,356,731	5,051,594	94.57	89.04
179	HK-38	568,721,717	6,030,682	94.74	89.41
180	HK-39	386,809,131	4,098,070	94.74	89.41
181	HK-40	378,884,554	4,023,618	94.62	89.18
182	HK-41	192,772,543	2,050,964	95.08	89.93
183	HK-42	221,321,662	2,341,824	94.84	89.55
184	HK-43	186,347,434	1,973,358	94.69	89.36
185	HK-44	313,105,671	3,366,356	94.86	89.45
186	HK-45	215,053,426	2,278,130	94.44	88.87
187	HK-46	404,037,648	4,285,394	94.37	88.74
188	HK-47	119,528,116	1,267,764	94.55	89.06
189	HK-49	302,833,966	3,207,638	94.83	89.56
190	HK-50	264,313,179	2,796,832	94.68	89.25
191	HK25-165	240,881,197	2,570,908	94.41	88.73
192	HK25-78	256,345,285	2,712,006	94.57	89.07
