# Supplementary material for: Identification of Loci Governing Agronomic Traits and Mutation Hotspots via a GBS-Based Genome-Wide Association Study in a Soybean Mutant Diversity Pool
Source: Int J Mol Sci. 2022 Sep 9;23(18):10441. doi: 10.3390/ijms231810441 (PMC9499481; doi:10.3390/ijms231810441)
Supplement: Supplementary file 1 [file ijms-23-10441-s001.zip › Table S3.pdf]

Table S3. Variant calling for the mapped reads.

Lines	Progressed Reads	Mapped Reads		Lines	Progressed Reads	Mapped Reads	
KAS523-7	7,649,550	7,317,569	95.66%	DP-028	10,441,632	10,233,725	98.01%
94Seori	3,903,690	3,706,390	94.95%	DP-029	3,899,686	3,716,598	95.31%
BS-25	7,547,426	7,301,067	96.74%	DP-046	4,114,452	3,860,853	93.84%
BS-63	3,275,656	3,153,291	96.26%	DP-048	1,948,254	1,860,717	95.51%
BS-73	6,821,852	6,674,374	97.84%	DP-051	7,443,956	7,202,406	96.76%
BS-74	9,307,644	9,145,850	98.26%	DP-052	7,406,186	7,167,732	96.78%
BS-84	8,899,140	8,605,623	96.70%	DP-053	7,231,542	6,772,244	93.65%
BangSa	10,288,844	7,836,341	76.16%	DP-054	4,196,558	3,984,055	94.94%
DanBaek	6,642,424	6,306,001	94.94%	DP-055	9,988,236	9,690,380	97.02%
DB-003	8,963,300	8,660,583	96.62%	DP-056	9,310,106	8,978,557	96.44%
DB-004	7,623,078	7,384,225	96.87%	DP-057	9,590,708	9,359,069	97.58%
DB-005	6,924,202	6,715,125	96.98%	DP-059	3,339,232	3,239,472	97.01%
DB-006	3,355,652	3,109,835	92.67%	DP-060	8,054,884	7,603,362	94.39%
DB-007	3,122,512	2,987,735	95.68%	DP-061	10,272,800	10,091,983	98.24%
DB-008	3,910,016	3,804,717	97.31%	DP-062	6,963,660	6,728,068	96.62%
DB-009	5,010,838	4,760,020	94.99%	DP-079	6,195,362	6,033,920	97.39%
DB-010	3,952,226	3,871,036	97.95%	DP-080	8,634,364	8,454,757	97.92%
DB-016	2,779,016	2,660,020	95.72%	DP-081	11,155,920	10,408,673	93.30%
DB-019	6,737,314	6,560,857	97.38%	DP-083	9,085,478	8,883,045	97.77%
DB-024	2,181,262	2,072,247	95.00%	DP-084	2,361,404	2,284,848	96.76%
DB-026	2,780,794	2,723,714	97.95%	DP-085	7,718,404	7,421,990	96.16%
DB-027	5,208,832	5,057,077	97.09%	DP-086	3,599,386	3,473,676	96.51%
DB-029	3,713,012	3,494,943	94.13%	DP-087	5,658,186	5,468,375	96.65%
DB-030	5,710,576	5,559,493	97.35%	DP-090	3,932,926	3,815,963	97.03%
DB-031	5,959,672	5,752,698	96.53%	DP-091	2,923,242	2,876,842	98.41%
DB-033	2,570,600	2,487,807	96.78%	DP-092	8,248,144	8,132,299	98.60%
DB-034	4,334,532	4,174,175	96.30%	DP-093	9,040,980	8,438,988	93.34%
DB-035	1,377,770	1,341,240	97.35%	DP-094	2,897,298	2,806,311	96.86%
DB-036	3,831,858	3,753,684	97.96%	DP-095	8,236,086	7,903,090	95.96%
DB-037	2,058,634	2,011,489	97.71%	DP-097	2,900,834	2,859,539	98.58%
DB-038	2,429,596	2,355,853	96.96%	DP-106	4,731,178	4,561,792	96.42%

DB-039	2,631,398	2,587,594	98.34%	DP-111	6,318,296	6,113,982	96.77%
DB-040	6,503,692	6,124,222	94.17%	DP-114	8,550,804	8,357,630	97.74%
DB-041	4,609,410	4,461,350	96.79%	DP-117	3,814,786	3,623,279	94.98%
DB-044	5,930,368	5,831,911	98.34%	DP-120	6,507,376	6,374,922	97.96%
DB-045	6,666,282	6,477,628	97.17%	DP-121	1,645,238	1,533,453	93.21%
DB-046	3,720,432	3,567,407	95.89%	DP-127	2,063,314	1,944,895	94.26%
DB-049	6,849,680	6,455,146	94.24%	DP-129	1,852,330	1,714,575	92.56%
DB-050	4,104,760	3,735,377	91.00%	DP-131	2,762,026	2,638,644	95.53%
DB-051	6,283,082	5,585,079	88.89%	DP-132	2,793,136	2,674,008	95.73%
DB-054	6,017,742	5,863,382	97.43%	DP-140	3,519,294	3,435,333	97.61%
DB-056	5,394,972	5,292,329	98.10%	DP-152	6,625,840	6,510,010	98.25%
DB-059	6,081,320	5,723,386	94.11%	DP-172	2,153,298	2,119,486	98.43%
DB-060	4,142,840	4,034,425	97.38%	DP-178	3,249,752	3,172,538	97.62%
DB-061	4,284,184	4,210,590	98.28%	DP-179	5,295,250	5,027,228	94.94%
DB-062	3,300,622	3,201,380	96.99%	DP-183	4,118,472	3,986,715	96.80%
DB-063	4,644,350	4,577,358	98.56%	DP-184	8,012,342	7,756,450	96.81%
DB-064	2,715,172	2,550,497	93.94%	DP-190	6,614,588	6,354,026	96.06%
DB-065	4,675,970	4,597,399	98.32%	DP-192	4,175,284	4,041,834	96.80%
DB-067	5,012,164	4,756,404	94.90%	DP-200	5,961,044	5,753,820	96.52%
DB-068	3,517,866	3,377,277	96.00%	HK-10	4,334,910	4,159,108	95.94%
DB-072	2,159,018	2,106,532	97.57%	HK-11	7,343,738	7,206,664	98.13%
DB-073	1,291,784	1,260,184	97.55%	HK-12	3,738,022	3,653,500	97.74%
DB-074	1,390,340	1,294,715	93.12%	HK-13	6,396,266	6,276,452	98.13%
DB-075	22,656	20,001	88.28%	HK-14	7,448,398	7,159,545	96.12%
DB-076	5,893,780	5,740,853	97.41%	HK-15	11,360,994	10,678,459	93.99%
DB-078	5,333,564	5,031,817	94.34%	HK-17	6,270,054	6,116,334	97.55%
DB-079	1,657,188	1,567,242	94.57%	HK-18	4,039,860	3,893,281	96.37%
DB-080	5,529,720	5,167,097	93.44%	HK-19	4,621,662	4,529,141	98.00%
DB-083	28,150	26,459	93.99%	HK-1	6,735,810	6,339,151	94.11%
DB-085	2,771,102	2,635,949	95.12%	HK-20	5,461,650	5,288,946	96.84%
DB-086	4,248,948	4,080,749	96.04%	HK-23	5,906,542	5,768,681	97.67%
DB-087	1,858,618	1,827,845	98.34%	HK-24	2,109,692	2,073,595	98.29%
DB-088	1,550,036	1,391,831	89.79%	HK25-165	2,570,908	2,355,098	91.61%

DB-089	1,697,242	1,643,407	96.83%	HK25-78	2,712,006	2,584,958	95.32%
DB-090	4,099,704	3,996,314	97.48%	HK-25	5,357,086	5,217,652	97.40%
DB-091	1,670,114	1,602,795	95.97%	HK-27	4,808,426	4,667,993	97.08%
DB-092	3,542,512	3,455,655	97.55%	HK-28	4,217,144	4,156,750	98.57%
DB-093	1,219,316	1,186,058	97.27%	HK-29	2,323,074	2,270,949	97.76%
DaePung	2,596,786	2,522,958	97.16%	HK-2	3,576,926	3,421,503	95.65%
DP-009	2,569,880	2,474,907	96.30%	HK-30	1,483,822	1,435,432	96.74%
DP-012	2,223,770	2,118,703	95.28%	HK-31	2,073,644	1,996,775	96.29%
DP-027	1,782,038	1,686,344	94.63%	HK-32	13,098	12,317	94.04%
JS-D01	7,165,048	6,727,981	93.90%	HK-33	7,246,082	6,919,317	95.49%
JS-D02	8,628,962	8,401,240	97.36%	HK-34	5,859,472	5,568,882	95.04%
JS-S	7,391,774	7,078,040	95.76%	HK-36	2,396,952	2,308,766	96.32%
JoseangSeori	1,857,636	1,819,037	97.92%	HK-37	5,051,594	4,835,064	95.71%
KAS360-22	5,195,146	5,038,048	96.98%	HK-38	6,030,682	5,891,383	97.69%
PD-D01	8,377,176	7,954,301	94.95%	HK-39	4,098,070	3,850,452	93.96%
PD-D02	7,260,034	7,006,109	96.50%	HK-3	2,942,544	2,798,506	95.10%
PD-D03	5,564,744	5,443,282	97.82%	HK-40	4,023,618	3,891,085	96.71%
PD-D04	2,595,514	2,491,578	96.00%	HK-41	2,050,964	1,975,878	96.34%
PD-D05	5,608,760	5,494,435	97.96%	HK-42	2,341,824	2,245,563	95.89%
PD-D06	2,788,036	2,740,730	98.30%	HK-43	1,973,358	1,930,209	97.81%
PD-D07	7,262,964	6,914,538	95.20%	HK-44	3,366,356	2,885,630	85.72%
PD-I01	4,531,986	4,439,799	97.97%	HK-45	2,278,130	2,235,395	98.12%
PD-I02	3,004,114	2,946,054	98.07%	HK-46	4,285,394	4,178,785	97.51%
PD-I03	8,214,314	8,097,931	98.58%	HK-47	1,267,764	1,240,580	97.86%
PD-I04	7,530,858	7,431,193	98.68%	HK-49	3,207,638	3,150,332	98.21%
PD-I05	4,006,582	3,943,296	98.42%	HK-4	3,678,100	3,465,298	94.21%
PD-I06	6,725,074	6,632,468	98.62%	HK-50	2,796,832	2,752,962	98.43%
PD-I07	3,605,464	3,474,416	96.37%	HK-5	7,742,168	7,283,375	94.07%
PalDal	6,872,236	6,650,131	96.77%	HK-6	6,115,882	5,826,048	95.26%
KAS523-7-Ru	4,581,976	4,407,956	96.20%	HK-8	5,305,742	5,067,657	95.51%
PD-I08-W	4,815,088	4,569,244	94.89%	HK-9	7,941,772	7,505,106	94.50%
KAS360-22-W	7,201,464	7,014,368	97.40%	HwangKeum	1,589,458	1,517,084	95.45%
