# Supplementary material for: Identification of Loci Governing Agronomic Traits and Mutation Hotspots via a GBS-Based Genome-Wide Association Study in a Soybean Mutant Diversity Pool
Source: Int J Mol Sci. 2022 Sep 9;23(18):10441. doi: 10.3390/ijms231810441 (PMC9499481; doi:10.3390/ijms231810441)
Supplement: Supplementary file 1 [file ijms-23-10441-s001.zip › Table S4.pdf]

Table S4. Filtered SNP data.

Lines	Total Hetero	Total Homo	SNP Ts/Tv Ratio	SNP Transition	SNP Transversion
DB-008	2092	6631	1.63	9521	5833
DB-009	2489	7089	1.61	10275	6392
DB-006	2335	6617	1.61	9596	5973
DB-007	2230	5991	1.59	8721	5491
DB-004	2728	8459	1.62	12158	7488
DB-005	3103	8277	1.62	12147	7510
DB-003	2836	8270	1.62	11976	7400
KAS360-22	2350	8181	1.57	11427	7285
HK25-165	1751	5223	1.66	7613	4584
DB-086	2069	6392	1.59	9113	5740
DB-087	1443	3666	1.63	5443	3332
DP-129	1530	3345	1.66	5132	3088
DB-085	1973	5366	1.68	7971	4734
DP-084	1672	4052	1.69	6138	3638
DB-083	7	45	2.03	65	32
DB-080	2955	7102	1.61	10576	6583
DP-080	2477	7351	1.6	10572	6607
HK-4	6380	4364	1.62	9335	5773
HK-5	4935	6279	1.58	10725	6768
DP-121	1131	2923	1.7	4391	2586
DP-120	2306	7065	1.62	10173	6263
DP-127	1870	3881	1.66	6012	3620
HK-1	2303	7731	1.58	10887	6878
DB-088	1230	2772	1.68	4245	2529
DB-089	1246	3288	1.68	4908	2914
DP-009	1992	5052	1.66	7550	4546
PalDal	2069	7036	1.64	10031	6110
HK-18	2162	6728	1.63	9670	5948
HK-19	2239	7141	1.59	10141	6380
HK-17	2427	8124	1.59	11473	7202
HK-14	2358	7884	1.58	11092	7034
HK-15	8556	4971	1.57	11288	7210
HK-12	2169	7053	1.59	10003	6272
DP-190	6420	5136	1.6	10261	6431
HK-10	3355	6171	1.59	9646	6051
HK-11	2374	7848	1.57	11048	7022
DB-019	2464	7474	1.61	10751	6661

DP-081	2559	7424	1.61	10736	6671
DP-062	3963	6624	1.65	10719	6492
DP-060	2585	7235	1.6	10507	6548
DP-061	2534	7419	1.62	10753	6619
DB-010	2227	6293	1.6	9121	5692
JS-D01	2394	8553	1.54	11823	7677
JS-D02	2460	8649	1.56	12041	7717
DP-093	5300	6187	1.6	10886	6788
DP-092	2753	7840	1.59	11323	7110
DP-097	1991	4993	1.65	7461	4516
DP-095	3020	7838	1.6	11507	7189
DP-094	2362	5160	1.64	7886	4796
DB-091	1347	3384	1.75	5168	2947
DB-090	2250	6607	1.62	9570	5894
DB-093	990	2264	1.78	3534	1984
BS-25	2478	8939	1.58	12456	7900
HK-3	1743	5388	1.65	7786	4733
DB-092	2169	5977	1.67	8830	5293
DP-152	2442	7523	1.62	10807	6681
DP-091	1884	5509	1.61	7963	4939
PD-I06	2558	8720	1.61	12347	7651
PD-I07	2541	7050	1.62	10293	6348
PD-I04	2539	8841	1.61	12469	7752
PD-I05	2239	7309	1.59	10360	6497
PD-I02	2186	6145	1.65	9005	5471
PD-I03	2514	8874	1.59	12436	7826
PD-I01	2363	7478	1.61	10679	6640
JS-S	2490	8770	1.55	12190	7840
DP-012	1820	4686	1.69	7030	4162
DP-079	2512	6980	1.61	10169	6303
DB-024	1692	4484	1.68	6678	3982
DB-026	1835	5220	1.66	7653	4622
DB-027	2283	6978	1.6	9990	6249
DB-029	2098	6549	1.6	9353	5843
DP-090	2077	6071	1.65	8862	5357
DB-078	2602	7151	1.62	10462	6442
DB-033	1779	5010	1.68	7389	4410
DP-140	2192	6113	1.62	8913	5505
HK-46	2220	7230	1.6	10265	6415
DP-200	2229	6632	1.62	9577	5916
HK-40	2093	6494	1.61	9303	5778

DB-037	1573	4042	1.69	6066	3591
DB-036	2141	6254	1.65	9111	5538
DB-035	1126	2697	1.69	4094	2426
DB-034	2104	7108	1.6	10055	6265
DP-048	1457	3804	1.63	5619	3446
HK-43	1280	3896	1.69	5694	3378
DB-031	2510	7859	1.61	11238	6990
DB-030	2401	7731	1.58	10951	6912
DP-046	2225	6516	1.66	9528	5729
DB-039	1794	5106	1.66	7500	4506
DB-038	1559	4583	1.71	6761	3964
DP-083	2402	7174	1.62	10349	6401
BangSa	2460	7823	1.64	11241	6865
HwangKeum	1168	3061	1.72	4613	2677
HK-8	7052	5370	1.59	10931	6861
PD-I08-W	2304	8093	1.6	11392	7098
DP-085	3414	6675	1.62	10371	6393
DP-172	1727	3911	1.6	5879	3670
JoseangSeori	1560	3910	1.72	5936	3444
DP-086	2097	6074	1.61	8784	5461
DP-178	3566	5065	1.65	8537	5159
DP-179	2651	7204	1.61	10511	6548
DP-087	9175	3636	1.62	10174	6273
DanBaek	2568	7935	1.66	11499	6939
BS-84	2460	9021	1.59	12590	7912
HK-6	2399	7862	1.59	11137	6986
DP-053	2526	7753	1.63	11167	6865
DP-052	2457	7729	1.65	11149	6766
DP-051	2476	7525	1.62	10838	6688
DP-057	2582	7603	1.62	11005	6783
DP-056	2511	7380	1.62	10668	6603
DP-055	2520	7375	1.63	10705	6565
DP-054	3357	5821	1.6	9221	5778
DP-192	5250	4698	1.63	9067	5579
DP-059	2071	5618	1.66	8296	5011
DB-040	2384	7378	1.65	10668	6472
DB-041	2262	6727	1.64	9770	5946
DB-046	2197	5671	1.65	8439	5100
HK-2	2045	6244	1.64	9032	5501
DB-044	2336	7163	1.65	10367	6295
DB-045	2277	7378	1.64	10592	6441

BS-74	2533	9048	1.59	12650	7979
DB-049	2402	7097	1.64	10321	6275
BS-73	2488	8817	1.56	12272	7850
KAS360-7-W	2333	8422	1.56	11699	7478
HK-37	2422	7377	1.61	10589	6587
HK-9	2543	7614	1.57	10869	6902
DP-027	1386	3397	1.72	5176	3004
HK-50	1683	5002	1.62	7229	4458
DP-183	4581	4756	1.63	8730	5363
DP-184	8435	4400	1.59	10591	6644
DP-028	2593	7520	1.61	10874	6759
DP-029	2864	6182	1.62	9414	5814
DB-051	2274	6863	1.67	10018	5982
DB-050	2098	6211	1.67	9077	5443
DB-054	2529	7318	1.64	10658	6507
DB-056	2586	7329	1.65	10730	6514
DB-059	2942	8197	1.61	11927	7409
BS-63	2052	6750	1.65	9683	5869
HK-13	2316	7649	1.58	10786	6828
HK-45	1662	4579	1.67	6769	4051
HK-44	1827	5349	1.67	7830	4695
HK-47	889	2370	1.76	3587	2042
DP-111	2411	6744	1.61	9805	6094
HK-41	1423	4272	1.65	6207	3760
DP-117	2254	5437	1.68	8224	4904
DP-114	2548	6970	1.59	10134	6354
HK-42	5166	3420	1.62	7426	4580
HK-49	1891	5980	1.6	8520	5331
DB-064	1998	5445	1.68	8076	4812
DB-065	2817	7480	1.64	11050	6727
94Seori	2183	6974	1.58	9886	6245
DB-067	2882	7279	1.69	10947	6493
DB-060	2618	6697	1.66	9988	6024
DB-061	2412	7172	1.63	10375	6381
DB-062	2677	6105	1.64	9248	5639
DB-063	2863	7364	1.62	10886	6705
DB-068	2502	6456	1.67	9631	5783
KAS523-7-Ru	2414	7928	1.61	11258	7012
HK25-78	3328	4554	1.62	7696	4740
HK-30	1023	2827	1.75	4250	2427
HK-31	1416	3889	1.66	5738	3456

HK-32	0	11	2.67	16	6
HK-33	2382	7631	1.59	10825	6819
HK-34	2361	7516	1.58	10642	6751
HK-36	1710	4693	1.61	6848	4248
DP-106	10178	3293	1.59	10288	6476
HK-38	2279	7557	1.57	10630	6763
HK-39	2132	6539	1.59	9334	5876
DaePung	1758	4550	1.66	6775	4083
DB-079	1399	3243	1.68	4941	2944
DB-016	2019	5373	1.62	7896	4869
HK-25	2323	7497	1.58	10596	6721
DB-073	1074	2428	1.81	3821	2109
DB-072	1967	4345	1.63	6604	4053
DB-076	2734	7375	1.59	10722	6762
DB-075	3	18	2.25	27	12
DB-074	1106	2367	1.75	3713	2127
PD-D03	4838	7051	1.61	11675	7265
PD-D02	2823	8154	1.6	11780	7351
PD-D01	2899	8495	1.6	12232	7657
PD-D07	6363	6190	1.6	11543	7200
PD-D06	1881	5740	1.62	8271	5090
PD-D05	2445	8217	1.61	11645	7234
PD-D04	1881	5534	1.69	8128	4821
KAS523-7	2492	9097	1.59	12694	7992
HK-23	7136	5299	1.58	10858	6876
HK-20	2695	7145	1.6	10463	6522
HK-27	2233	7315	1.61	10402	6461
DP-131	2060	4475	1.66	6870	4140
DP-132	2176	4756	1.67	7305	4383
HK-24	1565	4271	1.64	6285	3822
HK-29	1713	4514	1.67	6716	4025
HK-28	2121	6727	1.6	9581	5994
