# Supplementary material for: Identification of Loci Governing Agronomic Traits and Mutation Hotspots via a GBS-Based Genome-Wide Association Study in a Soybean Mutant Diversity Pool
Source: Int J Mol Sci. 2022 Sep 9;23(18):10441. doi: 10.3390/ijms231810441 (PMC9499481; doi:10.3390/ijms231810441)
Supplement: Supplementary file 1 [file ijms-23-10441-s001.zip › Table S5.pdf]

Table S5. Variant annotations and predicted effects.

SNPs (%)	Effect / Effect Impact / Functional Class
7,843 (20.80)	INTERGENIC MODIFIER
5,048 (13.40)	INTRON MODIFIER
N/A	CODON_CHANGE_PLUS_CODON_INSERTION MODERATE
N/A	STOP_LOST HIGH
12 (0.03)	STOP_LOST HIGH MISSENSE
21 (0.05)	SPLICE_SITE_DONOR HIGH
1,204 (3.20)	UTR_3_PRIME MODIFIER
5,618 (14.9)	UPSTREAM MODIFIER
N/A	FRAME_SHIFT HIGH
115 (0.30)	START_GAINED LOW
N/A	START_LOST HIGH
8 (0.02)	START_LOST HIGH MISSENSE
27 (0.07)	SPLICE_SITE_ACCEPTOR HIGH
N/A	CODON_CHANGE_PLUS_CODON_DELETION MODERATE
N/A	CODON_DELETION MODERATE
N/A	STOP_GAINED HIGH
210 (0.56)	STOP_GAINED HIGH NONSENSE
N/A	CODON_INSERTION MODERATE
4,674 (12.41)	DOWNSTREAM MODIFIER
731 (1.95)	UTR_5_PRIME MODIFIER
N/A	GENE_FUSION_REVERESE HIGH
5,193 (13.79)	SYNONYMOUS_CODING LOW SILENT
6 (0.02)	SYNONYMOUS_STOP LOW SILENT
6,961 (18.49)	NON_SYNONYMOUS_CODING MODERATE MISSENSE
2 (0.01)	NON_SYNONYMOUS_START LOW MISSENSE
37,673	Total
