# Supplementary material for: Identification of Loci Governing Agronomic Traits and Mutation Hotspots via a GBS-Based Genome-Wide Association Study in a Soybean Mutant Diversity Pool
Source: Int J Mol Sci. 2022 Sep 9;23(18):10441. doi: 10.3390/ijms231810441 (PMC9499481; doi:10.3390/ijms231810441)
Supplement: Supplementary file 1 [file ijms-23-10441-s001.zip › Table S6.pdf]

Table S6. Information regarding the barcode adapters used for the GBS analysis arranged according to the population structure.

No.	Lines	Barcode	No.	Lines	Barcode
1	DB-003	TCGTT	97	DP-129	GCTCTA
2	DB-004	GGTTGT	98	DP-127	TAGGAA
3	DB-005	CCAGCT	99	DP-046	ACTA
4	DB-006	TTCAGA	100	DP-028	CTCC
5	KAS523-7	CTCC	101	DP-027	TTCCTGGA
6	KAS523-7-Ru	TGCA	102	DP-012	TATCGGGA
7	DP-179	ATATGT	103	DP-009	GTGAGGGT
8	DP-178	ATCGTA	104	DB-090	CCATGGGT
9	BS-25	TCAC	105	DB-092	CGTGTGGT
10	BS-74	TCACC	106	DB-091	CGCGGAGA
11	BS-84	CTAGC	107	DP-054	GTAA
12	Bangsa	GATC	108	DB-093	GCTGTGGA
13	BS-73	CGCTT	109	DP-048	CAGA
14	BS-63	TGCGA	110	DP-051	AACT
15	JS-S	AACT	111	DP-052	GCGT
16	94seori	ACTA	112	DP-053	CGAT
17	Joseangseori	CAGA	113	DP-055	AGGC
18	JS-D01	GCGT	114	DP-056	GATC
19	JS-D02	CGAT	115	DP-060	CGCTT
20	HK-37	TGGTACGT	116	DP-061	TCACC
21	PD-D01	TTCTC	117	DP-057	TCAC
22	PD-I06	GGAAC	118	DP-059	TGCGA
23	PD-I02	ATTGA	119	DP-062	CTAGC
24	PD-I07	GTCAA	120	Daepung	GGATTGGT
25	PD-I03	CATCT	121	DP-200	GCCAGT
26	PD-I04	CCTAC	122	DP-117	GGTTGT
27	PD-I01	AGGAT	123	DP-114	TCGTT
28	PD-I05	GAGGA	124	DP-111	TACAT
29	PD-I08-W	TAATA	125	DP-079	ACAAA
30	PD-D02	AGCCC	126	DP-080	TTCTC
31	PD-D05	ACCGT	127	DP-083	GTATT
32	PD-D06	GCTTA	128	DP-081	AGCCC
33	PD-D04	CTGTA	129	DP-084	CTGTA
34	PD-D07	GGTGT	130	DP-085	ACCGT
35	PD-D03	GTATT	131	DP-086	GCTTA
36	Paldal	ACAAA	132	DP-097	GTCAA
37	KAS360-22-W	GTAA	133	DP-090	AGGAT
38	KAS360-22	AGGC	134	DP-091	ATTGA
39	HK-32	ACGACTAC	135	DP-120	CCAGCT
40	HK-1	GTACTT	136	DP-121	TTCAGA
41	HK-11	GAACTTC	137	DP-140	GAGATA
42	HK-14	AACGCCT	138	DB-051	GTCGATT
43	HK-33	TAGCATGC	139	DB-050	GGACCTA
44	HK-38	TCTCAGTC	140	DB-049	GAACTTC
45	HK-27	GCGGAAT	141	DB-046	GAATTCA
46	HK-25	CTACGGA	142	DB-045	AAAAGTT

47	HK-34	TAGGCCAT	143	DB-044	ATGAAAC
48	HK-9	AAAAGTT	144	DB-041	CTTGCTT
49	HK-18	ATTAATT	145	DB-040	TATTTTT
50	HK-40	CGCCTTAT	146	DB-037	GTTGAA
51	HK-39	CCGGATAT	147	DB-039	TGGCTA
52	HK-49	GGATTGGT	148	DB-038	TAACGA
53	HK-13	GTCGATT	149	DB-036	GTACTT
54	HK-19	ATTGGAT	150	DB-064	CGGTAGA
55	HK-28	TAGCGGA	151	DB-063	CGCTGAT
56	HK-3	TAACGA	152	DB-062	CATAAGT
57	HK-20	CATAAGT	153	DB-061	ATTGGAT
58	HK-2	GTTGAA	154	DB-060	ATTAATT
59	HK-44	CCATGGGT	155	Danbaek	TACAT
60	HK-50	GTGAGGGT	156	DB-085	CCGGATAT
61	HK-41	AACCGAGA	157	DB-056	AATATGC
62	HK-45	CGCGGAGA	158	DB-054	AACGCCT
63	HK-36	TGCAAGGA	159	DB-027	ATATGT
64	HK-29	TCGAAGA	160	DB-026	ACCTAA
65	HK-24	CGGTAGA	161	DB-010	CTTCCA
66	HK-43	ACGTGGTA	162	DB-035	GGAAGA
67	HK-31	TGCTGGA	163	DB-030	CATCGT
68	HK-30	TCTGTGA	164	DB-029	ATCGTA
69	HK-10	GAATTCA	165	DB-031	CGCGGT
70	HK-47	GCTGTGGA	166	DB-034	GCCAGT
71	HK-25-78	TATCGGGA	167	DB-033	CTATTA
72	Hwangkeum	GGAAGA	168	DB-019	ATGCCT
73	HK-4	TGGCTA	169	DB-008	GCTCTA
74	HK-42	ACAGGGAA	170	DB-024	AGTGGA
75	HK-12	GGACCTA	171	DB-009	CCACAA
76	HK-17	ACGTGTT	172	DB-016	GAGATA
77	HK-6	CTTGCTT	173	DB-007	TAGGAA
78	HK-46	CGTGTGGT	174	DB-065	CTACGGA
79	HK-5	TATTTTT	175	DB-068	TAGCGGA
80	HK-23	CGCTGAT	176	DB-067	GCGGAAT
81	HK-8	ATGAAAC	177	DP-106	TAATA
82	HK-15	AATATGC	178	DB-072	TCGAAGA
83	HK-25-165	TTCCTGGA	179	DB-073	TCTGTGA
84	DP-192	CTATTA	180	DB-074	TGCTGGA
85	DP-190	CGCGGT	181	DB-076	TAGCATGC
86	DP-184	CATCGT	182	DB-078	TAGGCCAT
87	DP-087	GGTGT	183	DB-080	TGGTACGT
88	DP-092	CATCT	184	DB-075	ACGACTAC
89	DP-093	CCTAC	185	DB-083	TCTCAGTC
90	DP-094	GAGGA	186	DB-079	TGCAAGGA
91	DP-095	GGAAC	187	DB-086	CGCCTTAT
92	DP-152	ATGCCT	188	DB-087	AACCGAGA
93	DP-029	TGCA	189	DB-089	ACGTGGTA
94	DP-183	ATCGTA	190	DB-088	ACAGGGAA
95	DP-132	CTTCCA	191	DP-172	AGTGGA
6	DP-131	CCACAA	192	DB-059	ACGTGTT
